# Supplementary material for: Genomic footprint of a shared Type 5 prophage in “Candidatus Liberibacter asiaticus” and “Candidatus Liberibacter africanus,” two destructive bacterial pathogens of citrus Huanglongbing
Source: Appl Environ Microbiol. 2025 Oct 15;91(11):e01072-25. doi: 10.1128/aem.01072-25 (PMC12628843; doi:10.1128/aem.01072-25)
Supplement: Supplemental material — Figures S1 to S5; Tables S1 to S9. [file aem.01072-25-s0001.docx]

**SUPPLEMENTAL MATERIAL**

**Genomic footprint of a shared Type 5 prophage in ‘*Candidatus* Liberibacter asiaticus’ and ‘*Candidatus* Liberibacter africanus’,** **two destructive bacterial pathogens of citrus huanglongbing**

Frédéric Labbé^1^*, Claudine Boyer^1^, Fernando Clavijo-Coppens^1^, Blandine Benoist^1^, Patrick Turpin^1^, Santatra Ravelomanantsoa^2^, Olivier Pruvost^1^

1 CIRAD, UMR PVBMT, F-97410 Saint Pierre, La Réunion, France

2 FOFIFA, National Center of Applied Research for Rural Development, Department of Agricultural Research (DRA), Antananarivo, Madagascar

*Correspondence: F. Labbé, Email: frederic.labbe@cirad.fr. Address: CIRAD, UMR PVBMT, Pôle de Protection des Plantes, 7, chemin de l’Irat, 97410 Saint Pierre, La Réunion, France


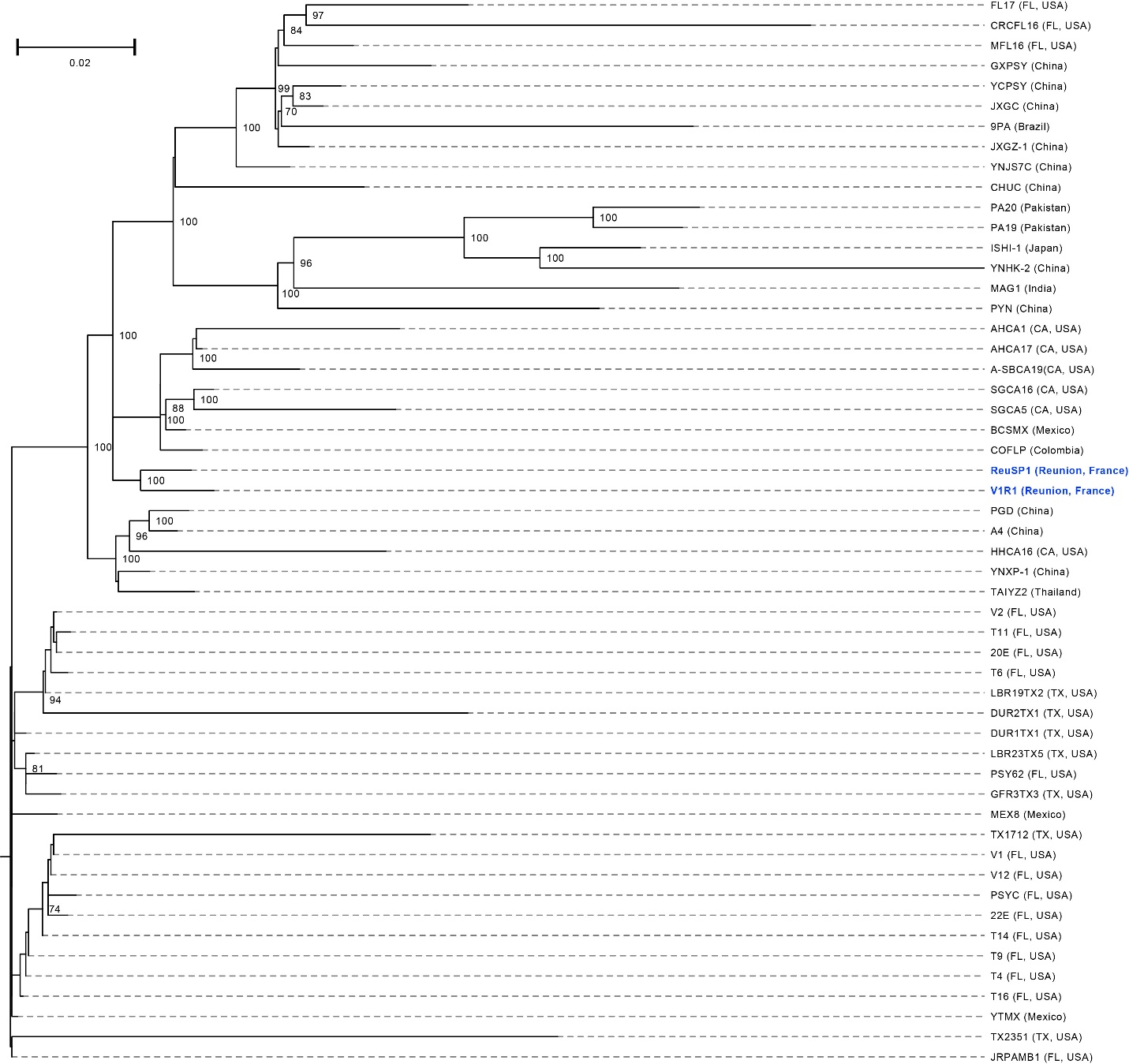


**FIG S1** **Maximum likelihood phylogenetic tree of the ‘*Candidatus* Liberibacter asiaticus’ (CLas) whole-genomes from public databases.** Support is shown for bootstrap values greater than 70. The CLas genomes from Réunion are highlighted in blue.


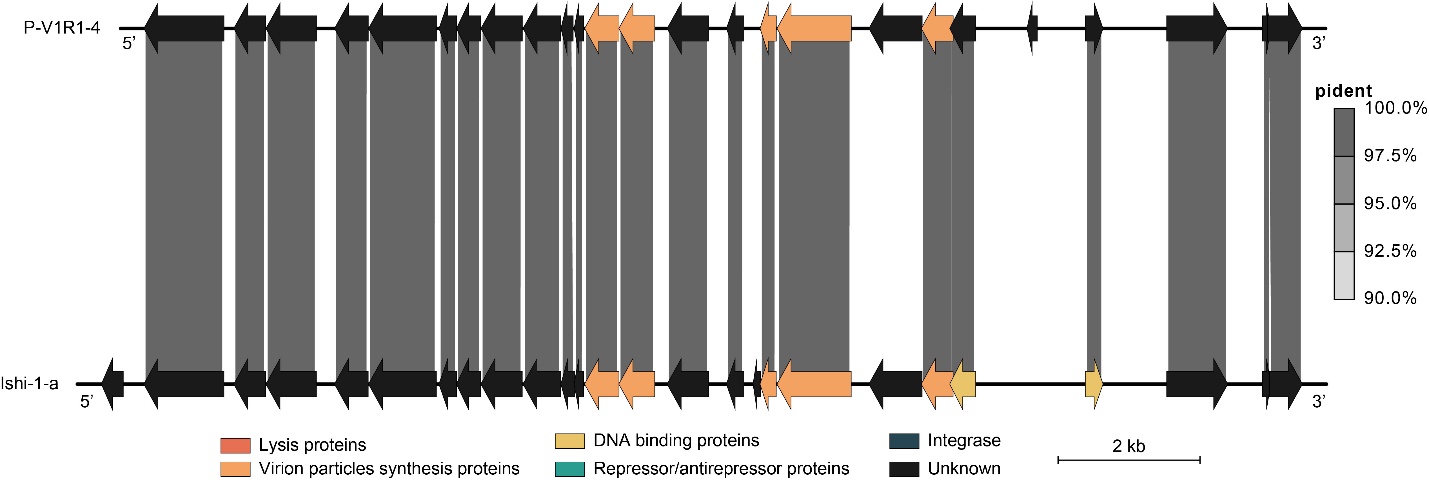


**FIG S2** **Genomic alignment map of two CLas Type 4 remnant prophages, *i.e.,* P-V1R1-4 and Ishi-1-a.** The predicted coding sequences (CDS) are indicated by arrows of different colors. The CDSs encoding proteins with a percentage of identity (pident) > 90%, a span > 80%, and e-value < 1e^-3^ are linked by light gray shadings.

**
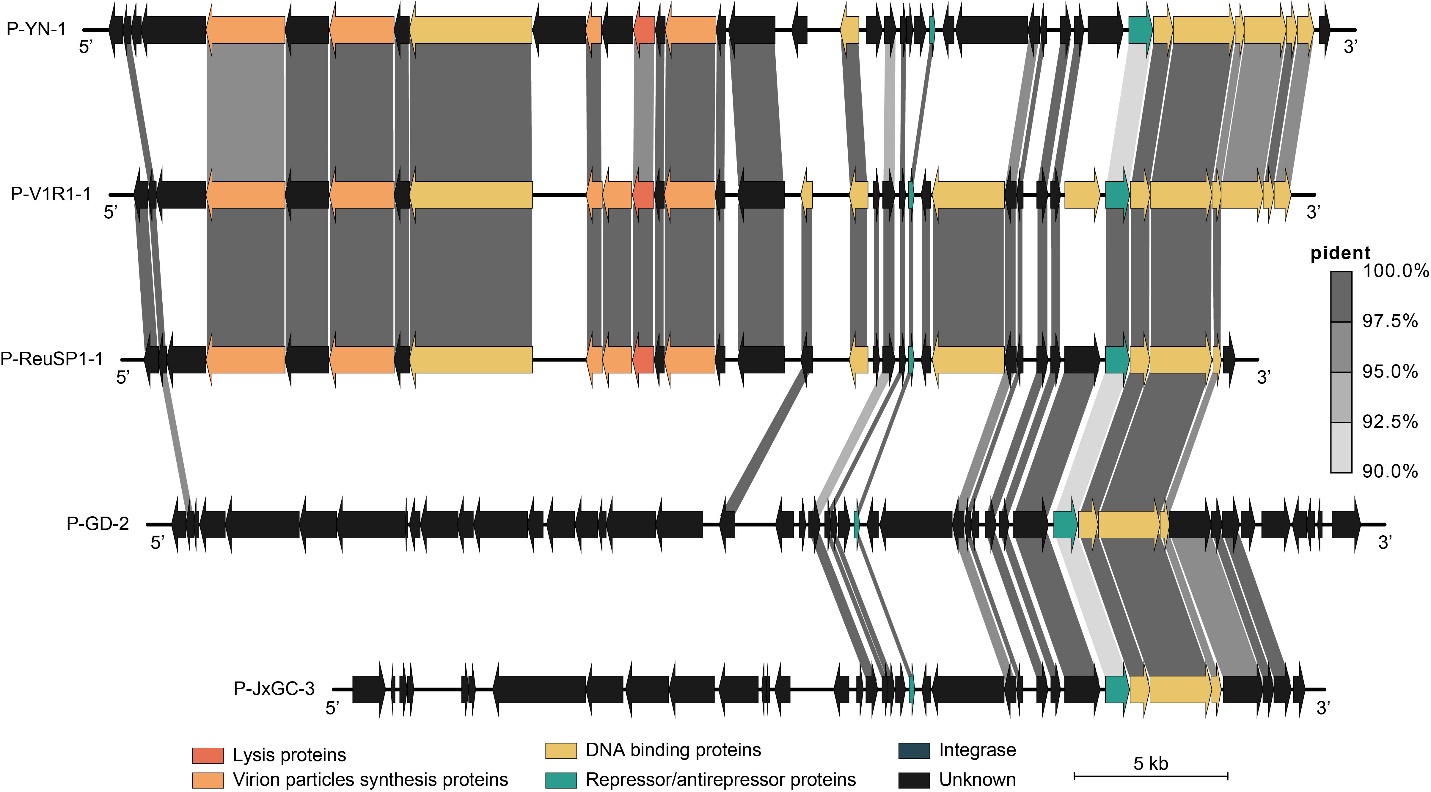
**

**FIG S3** **Genomic alignment map of the Type 1 CLas prophage P-V1R1-1 and four CLas prophages, *i.e.,* P-YN-1 (Type 1), P-ReuSP1-1, P-GD-2 (Type 2), and P-JXGC-3 (Type 3).** The predicted coding sequences (CDS) are indicated by arrows of different colors. The CDSs encoding proteins with a percentage of identity (pident) > 90%, a span > 80%, and e-value < 1e^-3^ are linked by light gray shadings.

**
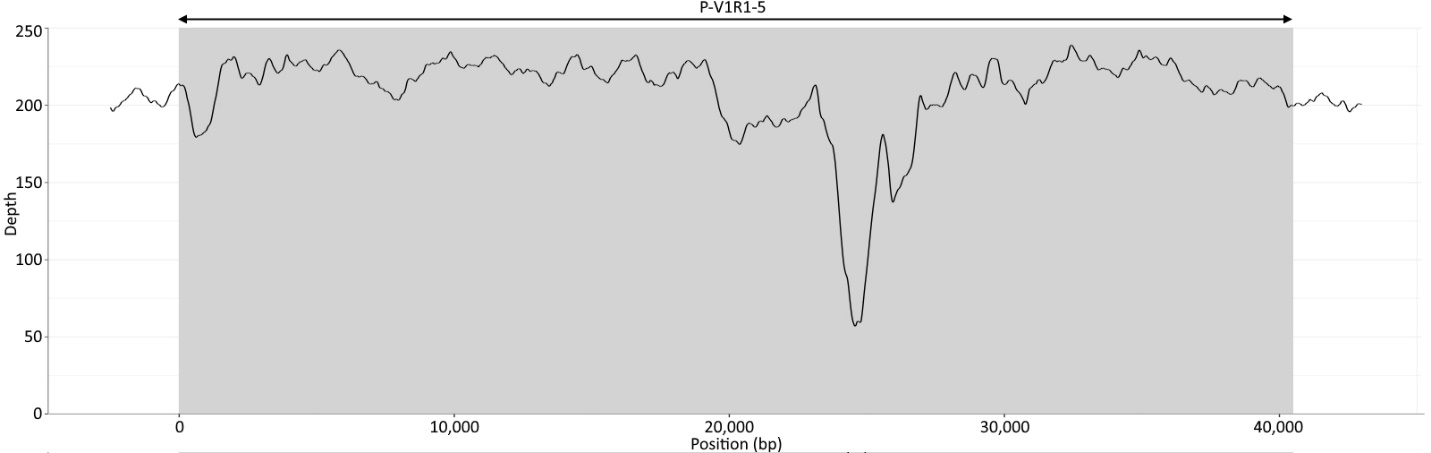
**

**FIG S4** **Type 5 CLas prophage P-V1R1-5 reads mapping.** Read depth (*i.e.,* the average number of mapped reads) per 1 kb non-overlapping windows across, and around (± 2,500 bp), the Type 5 CLas prophage P-V1R1-5.


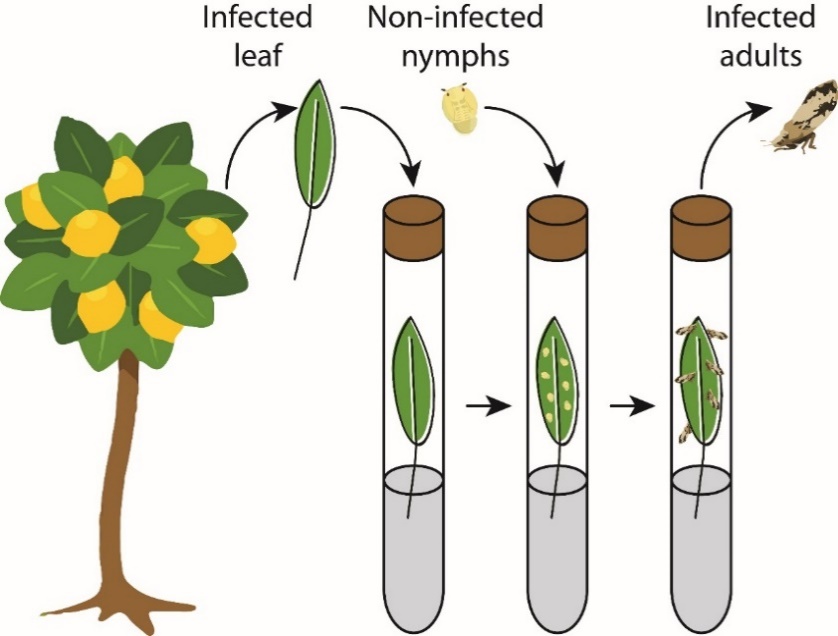


**FIG S5** **HLB acquisition assays (adapted from Reynaud *et al.* 2022).** During the acquisition access period, approximately 40 psyllid nymphs (from laboratory colonies) at the 3^rd^ to 4^th^ instar stage were introduced into tubes containing CLas-infected detached leaves from young field-collected plants. The petiole of each leaf was inserted into an Eppendorf tube filled with Murashige and Skoog (MS) medium, itself inserted in a glass tube closed by a fine mesh. The nymphs were left to feed and develop during 14 days on the infected leaves inside the tubes, *i.e.,* until the adults emerged. Newly emerged AfCP adults were transferred on healthy Volkamer lemon (*C. limonia* Osbeck ‘Volkameriana’) detached receptor leaves for 21 days to allow bacterial multiplication and were then preserved at -80°C for further tests.

**Table S1** Information about the *Candidatus* Liberibacter genomes used in this study. ǂ genomes used for the phylogeny analysis. * genomes used for the prophage comparisons. § WGS project or GenBank assembly number were provided when the GenBank accession number was not available. Abbreviations: CLas = ‘*Candidatus* Liberibacter asiaticus’, CLaf = ‘*Ca.* Liberibacter africanus’, CLam = ‘*Ca.* Liberibacter americanus’, CLso = *‘Ca.* Liberibacter solanacearum’, and CLeu = ‘*Ca.* Liberibacter europaeus’.

| **Strain** | **GenBank Accession Number** | **Species** | **Province or State** | **Country** | **Assembly size (Mb)** | **Reference** |
| --- | --- | --- | --- | --- | --- | --- |
| 9PAǂ | JABDRZ01§ | CLas | Sao Paulo | Brazil | 1.23188 | <https://doi.org/10.1094/PDIS-05-20-1018-A> |
| CHUCǂ | VTLV01§ | CLas | - | China | 1.20845 | <https://doi.org/10.1111/mpp.12925> |
| PGDǂ | CP100754.1 | CLas | Guangdong | China | 1.23022 | <https://doi.org/10.1128/spectrum.00754-23> |
| A4ǂ | CP010804.2 | CLas | Guangdong | China | 1.23025 | <https://doi.org/10.1128/genomea.00273-14> |
| YCPSYǂ | LIIM01§ | CLas | Guangdong | China | 1.23365 | <https://doi.org/10.1128/genomea.01316-15> |
| GXPSYǂ | CP004005.1 | CLas | Guangxi | China | 1.26824 | <https://doi.org/10.1128/genomea.00184-13> |
| JXGZ-1ǂ | VIQL01§ | CLas | Jiangxi | China | 1.21799 | <https://doi.org/10.1016/S2095-3119(20)63217-8> |
| JXGCǂ | CP019958.1 | CLas | Jiangxi | China | 1.22516 | <https://doi.org/10.1094/PHYTO-08-17-0282-R> |
| YNHK-2ǂ | WUUB01§ | CLas | Yunnan | China | 1.08957 | <https://doi.org/10.1016/S2095-3119(20)63217-8> |
| YNXP-1ǂ | VIGA01§ | CLas | Yunnan | China | 1.20707 | <https://doi.org/10.1016/S2095-3119(20)63217-8> |
| PYNǂ | CP100417.1 | CLas | Yunnan | China | 1.23126 | <https://doi.org/10.1128/spectrum.00754-23> |
| YNJS7Cǂ | QXDO01§ | CLas | Yunnan | China | 1.25899 | <https://doi.org/10.1128/mra.01413-18> |
| COFLPǂ | CP054558.1 | CLas | Municipio Dibulla | Colombia | 1.23164 | <https://doi.org/10.1094/PDIS-06-20-1249-A> |
| ReuSP1ǂ | CP061535.1 | CLas | La Reunion | France | 1.23000 | <https://doi.org/10.1094/PDIS-09-20-1998-A> |
| V1R1ǂ* | - | CLas | La Reunion | France | 1.27157 | This study |
| MAG1ǂ | JAJDMM01§ | CLas | - | India | 1.20743 | - |
| ISHI-1ǂ* | AP014595.1 | CLas | Okinawa | Japan | 1.19085 | <https://doi.org/10.1371/journal.pone.0106109> |
| BCSMXǂ | JAOPHS01§ | CLas | Baja California Sur | Mexico | 1.23000 | <https://doi.org/10.3389/fpls.2022.1052680> |
| MEX8ǂ | VTLU01§ | CLas | Mexicali | Mexico | 1.24313 | <https://doi.org/10.1111/mpp.12925> |
| YTMXǂ | JAOPHR01§ | CLas | Yucatan | Mexico | 1.23000 | <https://doi.org/10.3389/fpls.2022.1052680> |
| PA19ǂ | WOXD02§ | CLas | Multan | Pakistan | 1.22414 | <https://doi.org/10.1094/PDIS-12-19-2648-A> |
| PA20ǂ | WOUN02§ | CLas | Multan | Pakistan | 1.22623 | <https://doi.org/10.1094/PDIS-12-19-2648-A> |
| TAIYZ2ǂ | CP041385.1 | CLas | Songkhla | Thailand | 1.23062 | <https://doi.org/10.1094/PDIS-07-19-1520-A> |
| A-SBCA19ǂ | JADBIB01§ | CLas | California | USA | 1.18688 | <https://doi.org/10.3389/fmicb.2021.683481> |
| SGCA5ǂ | LMTO01§ | CLas | California | USA | 1.20138 | <https://doi.org/10.1128/genomea.01316-15> |
| HHCA16ǂ | VTLY01§ | CLas | California | USA | 1.20705 | <https://doi.org/10.1111/mpp.12925> |
| AHCA17ǂ | VNFL01§ | CLas | California | USA | 1.20862 | <https://doi.org/10.1094/PDIS-08-19-1735-A> |
| SGCA16ǂ | VTLZ01§ | CLas | California | USA | 1.20995 | <https://doi.org/10.1111/mpp.12925> |
| AHCA1ǂ | CP029348.1 | CLas | California | USA | 1.23375 | <https://doi.org/10.1094/PHYTO-06-18-0185-R> |
| MFL16ǂ | VTLX01§ | CLas | Florida | USA | 1.19922 | <https://doi.org/10.1111/mpp.12925> |
| CRCFL16ǂ | VTLW01§ | CLas | Florida | USA | 1.20828 | <https://doi.org/10.1111/mpp.12925> |
| T4ǂ | SRX13087740 | CLas | Florida | USA | 1.21451 | <https://doi.org/10.1094/PHYTO-02-22-0067-R> |
| T9ǂ | SRX13087760 | CLas | Florida | USA | 1.21492 | <https://doi.org/10.1094/PHYTO-02-22-0067-R> |
| T16ǂ | SRX13087752 | CLas | Florida | USA | 1.21495 | <https://doi.org/10.1094/PHYTO-02-22-0067-R> |
| 22Eǂ | SRX13087747 | CLas | Florida | USA | 1.21515 | <https://doi.org/10.1094/PHYTO-02-22-0067-R> |
| 20Eǂ | SRX13087766 | CLas | Florida | USA | 1.21548 | <https://doi.org/10.1094/PHYTO-02-22-0067-R> |
| T14ǂ | SRX13087780 | CLas | Florida | USA | 1.21630 | <https://doi.org/10.1094/PHYTO-02-22-0067-R> |
| FL17ǂ | JWHA01§ | CLas | Florida | USA | 1.22725 | <https://doi.org/10.1128/genomea.00169-15> |
| PSY62ǂ | CP001677.5 | CLas | Florida | USA | 1.22733 | <https://doi.org/10.1094/MPMI-22-8-1011> |
| PSYCǂ | SRX13087755 | CLas | Florida | USA | 1.23561 | <https://doi.org/10.1094/PHYTO-02-22-0067-R> |
| JRPAMB1ǂ | CP040636.1 | CLas | Florida | USA | 1.23717 | <https://doi.org/10.3390/microorganisms10030513> |
| T11ǂ | SRX13087741 | CLas | Florida | USA | 1.23913 | <https://doi.org/10.1094/PHYTO-02-22-0067-R> |
| V1ǂ | SRX13087758 | CLas | Florida | USA | 1.24007 | <https://doi.org/10.1094/PHYTO-02-22-0067-R> |
| V2ǂ | SRX13087757 | CLas | Florida | USA | 1.24025 | <https://doi.org/10.1094/PHYTO-02-22-0067-R> |
| T6ǂ | SRX13087773 | CLas | Florida | USA | 1.24102 | <https://doi.org/10.1094/PHYTO-02-22-0067-R> |
| V12ǂ | SRX13087781 | CLas | Florida | USA | 1.24147 | <https://doi.org/10.1094/PHYTO-02-22-0067-R> |
| LBR19TX2ǂ | VTMA01§ | CLas | Texas | USA | 1.20275 | <https://doi.org/10.1111/mpp.12925> |
| TX1712ǂ | QEWL01§ | CLas | Texas | USA | 1.20333 | <https://doi.org/10.1128/genomea.00554-18> |
| LBR23TX5ǂ | VTMB01§ | CLas | Texas | USA | 1.20348 | <https://doi.org/10.1111/mpp.12925> |
| DUR1TX1ǂ | VTLT01§ | CLas | Texas | USA | 1.20629 | <https://doi.org/10.1111/mpp.12925> |
| GFR3TX3ǂ | VTLR01§ | CLas | Texas | USA | 1.20932 | <https://doi.org/10.1111/mpp.12925> |
| DUR2TX1ǂ | VTLS01§ | CLas | Texas | USA | 1.21232 | <https://doi.org/10.1111/mpp.12925> |
| TX2351ǂ | MTIM01§ | CLas | Texas | USA | 1.25200 | <https://doi.org/10.1128/genomea.00170-17> |
| Ang37* | GCA_017869345.1§ | CLaf | - | Angola | 1.19198 | <https://doi.org/10.1094/PDIS-06-20-1392-PDN> |
| PTSAPSY* | GCA_001021085.1§ | CLaf | - | South Africa | 1.19223 | https://doi.org/10.1128/genomea.00733-15 |
| Zim* | GCF_040580775.1§ | CLaf | - | Zimbabwe | 1.18267 | <https://doi.org/10.1094/PDIS-05-24-1141-SC> |
| PW_SP* | GCA_000496595.1§ | CLam | Sao Paulo | Brazil | 1.19520 | https://doi.org/10.1094/MPMI-09-13-0292-R |
| CLso-ZC1* | NC_014774.1 | CLso | Texas | USA | 1.25828 | https://doi.org/10.1371/journal.pone.0019135 |
| ASNZ1* | PRJNA243548§ | CLeu | Canterbury | New Zealand | 1.33000 | https://doi.org/10.1128/genomea.00430-18 |

**Table S2** Predicted unique CDSs of the CLas V1R1 genome.

| **ID** | **Start** | **End** | **Length** | **Strand** | **Putative product** |
| --- | --- | --- | --- | --- | --- |
| DFOLPJBN_00143 | 158,289 | 158,660 | 124 | - | Hypothetical protein |
| DFOLPJBN_00147 | 160,622 | 161,218 | 199 | - | HTH cro/C1-type domain-containing protein |
| DFOLPJBN_00148 | 161,475 | 162,110 | 212 | + | PP_pnuc_1 domain-containing protein |
| DFOLPJBN_00149 | 162,165 | 162,518 | 118 | + | Rx_N domain-containing protein |
| DFOLPJBN_00153 | 164,014 | 165,753 | 580 | + | Hypothetical protein |
| DFOLPJBN_00155 | 166,334 | 166,927 | 198 | - | Trichohyalin-like |
| DFOLPJBN_00158 | 167,788 | 167,886 | 33 | + | Hypothetical protein |
| DFOLPJBN_00616 | 679,850 | 680,551 | 234 | - | Lipoprotein |
| DFOLPJBN_00802 | 868,788 | 869,564 | 259 | + | Collagen triple helix repeat |
| DFOLPJBN_00805 | 870,355 | 871,983 | 543 | - | Hypothetical protein |

**Table S3** Predicted CDSs of the P-V1R1-1 prophage.

| **ID** | **Start** | **End** | **Length** | **Strand** | **Putative product** |
| --- | --- | --- | --- | --- | --- |
| DFOLPJBN_00803 | 869,619 | 870,093 | 474 | - | Hypothetical protein |
| DFOLPJBN_00804 | 870,089 | 870,362 | 273 | - | Hypothetical protein |
| DFOLPJBN_00805 | 870,354 | 871,983 | 1,629 | - | Hypothetical protein |
| DFOLPJBN_00806 | 871,989 | 874,584 | 2,595 | - | Virion structural protein |
| DFOLPJBN_00807 | 874,580 | 876,017 | 1,437 | - | Hypothetical protein |
| DFOLPJBN_00808 | 876,034 | 878,158 | 2,124 | - | Head protein |
| DFOLPJBN_00809 | 878,154 | 878,670 | 516 | - | Hypothetical protein |
| DFOLPJBN_00810 | 878,650 | 882,694 | 4,044 | - | Exonuclease |
| DFOLPJBN_00812 | 884,447 | 884,975 | 528 | - | Tail protein |
| DFOLPJBN_00813 | 884,981 | 885,941 | 960 | - | Major head protein |
| DFOLPJBN_00814 | 885,966 | 886,668 | 702 | - | Protease |
| DFOLPJBN_00815 | 886,678 | 887,008 | 330 | - | Hypothetical protein |
| DFOLPJBN_00816 | 887,000 | 888,671 | 1,671 | - | Head-tail adaptor |
| DFOLPJBN_00817 | 888,667 | 889,000 | 333 | - | Hypothetical protein |
| DFOLPJBN_00819 | 889,408 | 890,944 | 1,536 | - | Hypothetical protein |
| DFOLPJBN_00820b | 891,478 | 891,865 | 387 | - | Terminase small subunit |
| DFOLPJBN_00821 | 893,065 | 893,683 | 618 | - | Transcriptional regulator |
| DFOLPJBN_00822 | 893,852 | 894,065 | 213 | + | Hypothetical protein |
| DFOLPJBN_00823 | 894,164 | 894,575 | 411 | + | Hypothetical protein |
| DFOLPJBN_00824 | 894,711 | 894,918 | 207 | + | Hypothetical protein |
| DFOLPJBN_00825 | 895,008 | 895,194 | 186 | + | Anti-repressor |
| DFOLPJBN_00826 | 895,423 | 895,735 | 312 | - | Hypothetical protein |
| DFOLPJBN_00827 | 895,782 | 898,152 | 2,370 | - | DNA primase |
| DFOLPJBN_00828 | 898,152 | 898,542 | 390 | - | Hypothetical protein |
| DFOLPJBN_00829 | 898,559 | 898,766 | 207 | - | Hypothetical protein |
| DFOLPJBN_00830 | 899,212 | 899,584 | 372 | + | Hypothetical protein |
| DFOLPJBN_00831b | 899,667 | 899,997 | 330 | + | Hypothetical protein |
| DFOLPJBN_00832 | 900,132 | 901,299 | 1,167 | + | Exonuclease |
| DFOLPJBN_00834 | 901,469 | 902,261 | 792 | + | Anti-repressor Ant |
| DFOLPJBN_00835 | 902,278 | 902,929 | 651 | + | Gp2.5-like ssDNA binding protein and ssDNA annealing protein |
| DFOLPJBN_00836 | 902,932 | 904,960 | 2,028 | + | DNA polymerase |
| DFOLPJBN_00837 | 904,956 | 905,268 | 312 | + | Endonuclease |
| DFOLPJBN_00838 | 905,252 | 906,638 | 1,386 | + | DNA helicase |
| DFOLPJBN_00839 | 906,630 | 906,990 | 360 | + | DNA ligase |
| DFOLPJBN_00840b | 906,991 | 907,552 | 561 | + | Guanylate kinase |

**Table S4** Comparisons of the P-V1R1-1 proteins against the proteins identified in known CLas prophages.

| **P-V1R1-1 ID** | **P-ReuSP1-1** | | |  |  | **P-YN-1 (Type 1)** | | | |  | **P-GD-2 (Type 2)** | | | |  | **P-JXGC-3 (Type 3)** | | | |
| --- | --- | --- | --- | --- | --- | --- | --- | --- | --- | --- | --- | --- | --- | --- | --- | --- | --- | --- | --- |
|  | **ID** | **Span** | **pident** | **evalue** |  | **ID** | **Span** | **pident** | **evalue** |  | **ID** | **Span** | **pident** | **evalue** |  | **ID** | **Span** | **pident** | **evalue** |
| DFOLPJBN_00803 | WP_157141276.1 | 0.99 | 100.00 | 2.41E-115 |  | SC1_gp005 | 0.99 | 82.17 | 6.54E-97 |  | SC2_gp250 | 0.99 | 98.73 | 7.33E-114 |  | PJXGC_gp11 | 0.43 | 25.00 | 3.10E+00 |
| DFOLPJBN_00804 | WP_015453071.1 | 0.99 | 100.00 | 3.10E-66 |  | SC1_gp010 | 0.99 | 100.00 | 3.76E-66 |  | SC2_gp010 | 0.99 | 98.89 | 5.34E-65 |  | PJXGC_gp16 | 0.25 | 34.78 | 9.00E-01 |
| **DFOLPJBN_00805** | **NA** | **0.60** | **93.54** | **0.00E+00** |  | **SC1_gp025** | **0.76** | **75.67** | **0.00E+00** |  | **SC2_gp020** | **0.34** | **79.12** | **1.98E-93** |  | **PJXGC_gp11** | **0.13** | **22.86** | **8.50E+00** |
| DFOLPJBN_00806 | WP_238556160.1 | 1.00 | 100.00 | 0.00E+00 |  | SC1_gp030 | 1.00 | 96.07 | 0.00E+00 |  | SC2_gp030 | 1.00 | 23.73 | 2.73E-33 |  | PJXGC_gp07 | 0.05 | 26.83 | 5.90E+00 |
| DFOLPJBN_00807 | WP_238556161.1 | 1.00 | 100.00 | 0.00E+00 |  | SC1_gp035 | 1.00 | 98.95 | 0.00E+00 |  | SC2_gp040 | 0.49 | 26.70 | 6.07E-16 |  | PJXGC_gp05 | 0.08 | 30.77 | 1.70E+00 |
| DFOLPJBN_00808 | WP_238556162.1 | 1.00 | 100.00 | 0.00E+00 |  | SC1_gp045 | 1.00 | 98.59 | 0.00E+00 |  | SC2_gp045 | 1.00 | 19.69 | 2.08E-23 |  | PJXGC_gp07 | 0.10 | 22.54 | 2.40E+00 |
| DFOLPJBN_00809 | WP_040215722.1 | 0.99 | 100.00 | 2.99E-127 |  | SC1_gp050 | 0.97 | 99.40 | 1.26E-123 |  | SC2_gp065 | 0.58 | 27.27 | 2.19E-06 |  | NA | NA | NA | NA |
| DFOLPJBN_00810 | WP_056928525.1 | 1.00 | 100.00 | 0.00E+00 |  | SC1_gp060 | 1.00 | 98.81 | 0.00E+00 |  | SC2_gp070 | 0.09 | 30.33 | 2.46E-09 |  | PJXGC_gp10 | 0.04 | 32.73 | 6.60E+00 |
| DFOLPJBN_00812 | WP_238556164.1 | 0.99 | 99.43 | 6.02E-132 |  | SC1_gp085 | 0.99 | 98.29 | 2.42E-129 |  | SC2_gp085 | 0.76 | 43.28 | 3.46E-31 |  | PJXGC_gp11 | 0.20 | 17.14 | 4.30E-01 |
| DFOLPJBN_00813 | WP_015453080.1 | 1.00 | 100.00 | 0.00E+00 |  | SC1_gp090 | 1.00 | 52.25 | 1.43E-119 |  | SC2_gp090 | 0.91 | 51.20 | 1.45E-98 |  | PJXGC_gp07 | 0.18 | 36.21 | 1.90E-01 |
| DFOLPJBN_00814 | WP_015453081.1 | 1.00 | 100.00 | 4.92E-177 |  | SC1_gp095 | 0.98 | 96.52 | 1.30E-168 |  | SC2_gp095 | 0.74 | 33.53 | 4.54E-25 |  | PJXGC_gp09 | 0.28 | 25.76 | 3.70E+00 |
| DFOLPJBN_00815 | WP_015453082.1 | 0.99 | 100.00 | 2.29E-81 |  | SC1_gp100 | 0.99 | 100.00 | 2.77E-81 |  | SC2_gp100 | 0.63 | 40.58 | 4.13E-16 |  | PJXGC_gp31 | 0.41 | 31.11 | 7.80E-01 |
| DFOLPJBN_00816 | WP_015453083.1 | 1.00 | 100.00 | 0.00E+00 |  | SC1_gp105 | 1.00 | 100.00 | 0.00E+00 |  | SC2_gp105 | 0.98 | 40.18 | 2.29E-128 |  | PJXGC_gp11 | 0.12 | 23.53 | 9.20E+00 |
| DFOLPJBN_00817 | WP_015453084.1 | 0.99 | 100.00 | 1.32E-77 |  | SC1_gp110 | 0.99 | 100.00 | 1.60E-77 |  | SC2_gp030 | 0.63 | 22.86 | 3.50E-01 |  | PJXGC_gp06 | 0.62 | 26.09 | 3.30E+00 |
| DFOLPJBN_00819 | WP_015453086.1 | 1.00 | 100.00 | 0.00E+00 |  | SC1_gp115 | 1.00 | 99.02 | 0.00E+00 |  | SC2_gp115 | 1.00 | 76.37 | 0.00E+00 |  | PJXGC_gp11 | 0.82 | 99.28 | 0.00E+00 |
| DFOLPJBN_00820b | WP_015453087.1 | 0.99 | 100.00 | 2.56E-94 |  | SC1_gp120 | 0.99 | 100.00 | 1.24E-94 |  | SC2_gp120 | 0.99 | 100.00 | 1.18E-94 |  | PJXGC_gp14 | 0.98 | 68.25 | 1.60E-62 |
| DFOLPJBN_00821 | WP_157140849.1 | 1.00 | 100.00 | 1.07E-155 |  | SC1_gp125 | 1.00 | 99.51 | 1.34E-155 |  | SC2_gp125 | 0.97 | 69.35 | 1.79E-100 |  | PJXGC_gp15 | 0.80 | 57.58 | 1.63E-64 |
| DFOLPJBN_00822 | WP_238556165.1 | 0.99 | 100.00 | 1.20E-51 |  | SC1_gp185 | 0.62 | 27.27 | 3.10E+00 |  | SC2_gp130 | 0.99 | 90.00 | 4.21E-45 |  | PJXGC_gp16 | 0.99 | 82.86 | 1.09E-41 |
| DFOLPJBN_00823 | WP_015453061.1 | 1.00 | 30.50 | 1.23E-11 |  | SC1_gp185 | 1.00 | 30.50 | 1.49E-11 |  | SC2_gp185 | 1.00 | 30.50 | 2.31E-11 |  | PJXGC_gp26 | 1.00 | 30.50 | 1.02E-11 |
| DFOLPJBN_00824 | WP_015453053.1 | 0.99 | 100.00 | 7.41E-48 |  | SC1_gp140 | 0.99 | 100.00 | 8.97E-48 |  | SC2_gp140 | 0.99 | 100.00 | 8.56E-48 |  | PJXGC_gp01 | 1.00 | 25.61 | 7.90E-01 |
| DFOLPJBN_00825 | NA | 0.98 | 100.00 | 9.20E-43 |  | SC1_gp155 | 0.98 | 100.00 | 1.11E-42 |  | SC2_gp155 | 0.98 | 100.00 | 1.06E-42 |  | PJXGC_gp21 | 0.98 | 100.00 | 7.63E-43 |
| DFOLPJBN_00826 | WP_040055341.1 | 0.99 | 99.03 | 1.20E-73 |  | SC1_gp160 | 1.00 | 82.40 | 8.06E-69 |  | SC2_gp160 | 1.00 | 82.40 | 7.68E-69 |  | PJXGC_gp22 | 0.99 | 89.32 | 2.02E-62 |
| DFOLPJBN_00827 | WP_238556167.1 | 1.00 | 100.00 | 0.00E+00 |  | SC1_gp165 | 1.00 | 88.23 | 0.00E+00 |  | SC2_gp165 | 1.00 | 86.71 | 0.00E+00 |  | PJXGC_gp23 | 1.00 | 90.62 | 0.00E+00 |
| DFOLPJBN_00828 | WP_109298656.1 | 0.99 | 100.00 | 3.89E-89 |  | SC1_gp170 | 0.99 | 95.35 | 3.53E-85 |  | SC2_gp170 | 0.99 | 95.35 | 1.42E-84 |  | PJXGC_gp24 | 0.99 | 99.23 | 9.25E-89 |
| DFOLPJBN_00829 | WP_015453060.1 | 0.99 | 100.00 | 4.57E-48 |  | SC1_gp175 | 0.99 | 100.00 | 5.53E-48 |  | SC2_gp175 | 0.99 | 100.00 | 5.28E-48 |  | PJXGC_gp25 | 0.99 | 100.00 | 3.79E-48 |
| DFOLPJBN_00830 | WP_238556166.1 | 1.00 | 29.79 | 1.31E-09 |  | SC1_gp135 | 1.00 | 31.85 | 2.45E-12 |  | SC2_gp135 | 1.00 | 31.85 | 2.34E-12 |  | PJXGC_gp17 | 1.00 | 31.85 | 1.68E-12 |
| DFOLPJBN_00831b | WP_012778343.1 | 0.99 | 100.00 | 3.20E-79 |  | SC1_gp190 | 0.97 | 100.00 | 1.57E-77 |  | SC2_gp190 | 0.97 | 100.00 | 1.50E-77 |  | PJXGC_gp27 | 0.97 | 100.00 | 1.08E-77 |
| **DFOLPJBN_00832** | **WP_157141068.1** | **1.00** | **88.66** | **0.00E+00** |  | **SC1_gp195** | **0.99** | **88.37** | **0.00E+00** |  | **SC2_gp195** | **0.99** | **88.11** | **0.00E+00** |  | **PJXGC_gp28** | **1.00** | **89.43** | **0.00E+00** |
| DFOLPJBN_00834 | WP_238556168.1 | 1.00 | 100.00 | 0.00E+00 |  | SC1_gp200 | 1.00 | 90.49 | 0.00E+00 |  | SC2_gp200 | 1.00 | 90.49 | 0.00E+00 |  | PJXGC_gp29 | 1.00 | 99.62 | 0.00E+00 |
| DFOLPJBN_00835 | WP_015453064.1 | 1.00 | 100.00 | 9.93E-167 |  | SC1_gp205 | 1.00 | 98.15 | 2.28E-163 |  | SC2_gp205 | 1.00 | 98.15 | 2.18E-163 |  | PJXGC_gp30 | 1.00 | 100.00 | 8.23E-167 |
| DFOLPJBN_00836 | WP_238556170.1 | 1.00 | 100.00 | 0.00E+00 |  | SC1_gp210 | 1.00 | 99.85 | 0.00E+00 |  | SC2_gp210 | 1.00 | 99.85 | 0.00E+00 |  | PJXGC_gp31 | 1.00 | 99.85 | 0.00E+00 |
| DFOLPJBN_00837 | WP_015453066.1 | 0.87 | 100.00 | 2.69E-66 |  | SC1_gp215 | 0.99 | 97.09 | 4.32E-75 |  | SC2_gp215 | 0.99 | 96.12 | 6.19E-75 |  | PJXGC_gp32 | 0.99 | 100.00 | 7.96E-78 |
| DFOLPJBN_00838 | WP_238556171.1 | 0.29 | 100.00 | 1.36E-94 |  | SC1_gp220 | 1.00 | 96.10 | 0.00E+00 |  | SC2_gp220 | 1.00 | 96.10 | 0.00E+00 |  | PJXGC_gp33 | 0.94 | 99.77 | 0.00E+00 |
| DFOLPJBN_00839 | WP_056928525.1 | 0.45 | 25.93 | 3.20E+00 |  | SC1_gp225 | 0.99 | 100.00 | 1.00E-89 |  | SC2_gp225 | 0.99 | 100.00 | 9.57E-90 |  | PJXGC_gp34 | 0.99 | 100.00 | 6.87E-90 |
| DFOLPJBN_00840b | WP_238556170.1 | 0.60 | 25.66 | 6.80E+00 |  | SC1_gp230 | 0.97 | 97.24 | 3.26E-135 |  | SC2_gp230 | 0.95 | 53.37 | 3.01E-68 |  | PJXGC_gp35 | 0.95 | 52.81 | 1.48E-67 |

**Table S5** Predicted CDSs of the P-V1R1-5 prophage.

| **ID** | **Start** | **End** | **Length** | **Strand** | **Putative product** |
| --- | --- | --- | --- | --- | --- |
| DFOLPJBN_00125 | 136,969 | 137,568 | 600 | + | Hypothetical protein |
| DFOLPJBN_00126 | 137,572 | 138,048 | 477 | - | Holin |
| DFOLPJBN_00127 | 138,045 | 138,302 | 258 | - | Phage protein |
| DFOLPJBN_00128 | 138,299 | 140,734 | 2,436 | - | Virion structural protein |
| DFOLPJBN_00129 | 140,734 | 141,888 | 1,155 | - | Phage protein |
| DFOLPJBN_00130 | 141,898 | 144,030 | 2,133 | - | Putative endolysin protein |
| DFOLPJBN_00131 | 144,036 | 144,485 | 450 | - | Hypothetical protein |
| DFOLPJBN_00132 | 144,485 | 147,406 | 2,922 | - | Exonuclease |
| DFOLPJBN_00133 | 147,406 | 149,154 | 1,749 | - | Tail assembly, connector, tail fibers |
| DFOLPJBN_00134 | 149,147 | 149,674 | 528 | - | Tail tubular protein |
| DFOLPJBN_00135 | 149,674 | 150,714 | 1,041 | - | Major capsid protein |
| DFOLPJBN_00136 | 150,738 | 151,457 | 720 | - | Regulatory protein RecX |
| DFOLPJBN_00137 | 151,471 | 151,797 | 327 | - | Phage protein |
| DFOLPJBN_00138 | 151,790 | 153,460 | 1,671 | - | Head-tail adaptor |
| DFOLPJBN_00139 | 153,460 | 153,783 | 324 | - | Hypothetical protein |
| DFOLPJBN_00140 | 153,776 | 154,882 | 1,107 | - | Terminase, large subunit |
| DFOLPJBN_00141 | 155,806 | 156,312 | 507 | - | Terminase small subunit |
| DFOLPJBN_00142 | 156,531 | 156,968 | 438 | - | Holliday junction resolvase |
| DFOLPJBN_00143 | 158,289 | 158,531 | 243 | - | Hypothetical protein |
| DFOLPJBN_00144 | 158,772 | 159,284 | 513 | + | Hypothetical protein involved in Toxin-Antitoxin complex |
| DFOLPJBN_00145 | 159,277 | 159,753 | 477 | + | Putative Repressor, Anti-repressor, complex |
| DFOLPJBN_00146 | 159,905 | 160,546 | 642 | - | Hypothetical protein |
| DFOLPJBN_00147 | 160,622 | 161,218 | 597 | - | Transcriptional regulator, contains XRE-family HTH domain |
| DFOLPJBN_00148 | 161,475 | 162,110 | 636 | + | Phage protein |
| DFOLPJBN_00149 | 162,165 | 162,518 | 354 | + | Phage protein |
| DFOLPJBN_00150 | 162,649 | 162,879 | 231 | + | Putative lipoprotein |
| DFOLPJBN_00151 | 162,955 | 163,761 | 807 | + | Bro-N family phage antirepressor |
| DFOLPJBN_00152 | 163,846 | 164,010 | 165 | + | Hypothetical protein |
| DFOLPJBN_00153 | 164,014 | 165,753 | 1,740 | + | Virion structural protein |
| DFOLPJBN_00154 | 165,750 | 166,073 | 324 | - | Phage protein |
| DFOLPJBN_00154b | 166,075 | 166,212 | 138 | - | Hypothetical protein |
| DFOLPJBN_00155 | 166,334 | 166,927 | 594 | - | Phage protein |
| DFOLPJBN_00156 | 166,920 | 167,138 | 219 | - | Hypothetical protein |
| DFOLPJBN_00157 | 167,135 | 167,401 | 267 | - | Phage protein |
| DFOLPJBN_00159 | 167,905 | 168,366 | 462 | + | Exonuclease |
| DFOLPJBN_00160 | 168,377 | 169,207 | 831 | + | Putative RecA protein |
| DFOLPJBN_00161 | 169,223 | 169,834 | 612 | + | DNA-binding protein |
| DFOLPJBN_00162 | 169,838 | 171,436 | 1,599 | + | DNA helicase |
| DFOLPJBN_00163 | 171,433 | 172,152 | 720 | + | CRISPR-associated exonuclease |
| DFOLPJBN_00164 | 172,139 | 174,235 | 2,097 | + | RecA-family ATPase |
| DFOLPJBN_00165 | 174,252 | 174,482 | 231 | + | Hypothetical protein |
| DFOLPJBN_00166 | 174,568 | 174,936 | 369 | + | Hypothetical protein |
| DFOLPJBN_00167 | 174,977 | 175,297 | 321 | + | DNA ligase |
| DFOLPJBN_00168 | 175,300 | 175,851 | 552 | + | Guanylate kinase |
| DFOLPJBN_00169 | 175,848 | 176,021 | 174 | + | Hypothetical protein |
| DFOLPJBN_00170 | 176,147 | 177,046 | 900 | + | Integrase |

**Table S6** Comparisons of the P-V1R1-5 proteins against the proteins identified in known CLaf prophages.

| **P-V1R1-5 ID** | **P-Zim-1** | | | |  | **P-Ang37-1** | | | |  | **P-PTSAPSY-1** | | | |
| --- | --- | --- | --- | --- | --- | --- | --- | --- | --- | --- | --- | --- | --- | --- |
|  | **Coord** | **Span** | **pident** | **evalue** |  | **Coord** | **Span** | **pident** | **evalue** |  | **Coord** | **Span** | **pident** | **evalue** |
| **DFOLPJBN_00125** | 473026-473560 | 0.89 | 100.00 | 1.80E-130 |  | **448882-449266** | **0.64** | **88.19** | **3.00E-78** |  | 448815-449349 | 0.89 | 100.00 | 1.77E-130 |
| **DFOLPJBN_00126** | **472546-473023** | **0.99** | **89.87** | **3.35E-105** |  | **457097-459104** | **0.42** | **22.73** | **1.30E+00** |  | **449352-449829** | **0.99** | **89.87** | **3.28E-105** |
| **DFOLPJBN_00127** | 472292-472550 | 0.99 | 100.00 | 9.24E-63 |  | **449742-449940** | **0.76** | **86.15** | **6.28E-39** |  | 449825-450083 | 0.99 | 100.00 | 9.06E-63 |
| DFOLPJBN_00128 | 469860-472296 | 1.00 | 99.63 | 0.00E+00 |  | 449997-452433 | 1.00 | 99.75 | 0.00E+00 |  | 450079-452515 | 1.00 | 99.75 | 0.00E+00 |
| DFOLPJBN_00129 | 468706-469861 | 1.00 | 98.44 | 0.00E+00 |  | 452432-453587 | 1.00 | 99.48 | 0.00E+00 |  | 452514-453669 | 1.00 | 98.96 | 0.00E+00 |
| DFOLPJBN_00130 | 466564-468697 | 1.00 | 99.58 | 0.00E+00 |  | 453596-455729 | 1.00 | 99.72 | 0.00E+00 |  | 453678-455811 | 1.00 | 99.86 | 0.00E+00 |
| DFOLPJBN_00131 | 466109-466559 | 0.99 | 100.00 | 3.22E-109 |  | 455734-456184 | 0.99 | 98.66 | 6.03E-108 |  | 455816-456266 | 0.99 | 100.00 | 3.16E-109 |
| **DFOLPJBN_00132** | 463188-466110 | 1.00 | 99.49 | 0.00E+00 |  | **457097-459104** | **0.68** | **97.30** | **0.00E+00** |  | 456265-459187 | 1.00 | 99.49 | 0.00E+00 |
| DFOLPJBN_00133 | 461440-463189 | 1.00 | 100.00 | 0.00E+00 |  | 459103-460852 | 1.00 | 99.31 | 0.00E+00 |  | 459186-460935 | 1.00 | 100.00 | 0.00E+00 |
| DFOLPJBN_00134 | 460920-461448 | 0.99 | 100.00 | 4.14E-130 |  | 460844-461372 | 0.99 | 98.86 | 2.67E-129 |  | 460927-461455 | 0.99 | 100.00 | 4.05E-130 |
| DFOLPJBN_00135 | 459880-460921 | 1.00 | 99.71 | 0.00E+00 |  | 461371-462412 | 1.00 | 99.71 | 0.00E+00 |  | 461454-462495 | 1.00 | 99.71 | 0.00E+00 |
| DFOLPJBN_00136 | 459137-459857 | 1.00 | 100.00 | 0.00E+00 |  | 462435-463155 | 1.00 | 97.91 | 9.75E-179 |  | 462518-463238 | 1.00 | 100.00 | 0.00E+00 |
| DFOLPJBN_00137 | 458797-459124 | 0.99 | 100.00 | 5.57E-80 |  | 463168-463495 | 0.99 | 100.00 | 5.38E-80 |  | 463251-463578 | 0.99 | 100.00 | 5.45E-80 |
| DFOLPJBN_00138 | 457134-458805 | 1.00 | 99.64 | 0.00E+00 |  | 463487-465158 | 1.00 | 99.28 | 0.00E+00 |  | 463570-465241 | 1.00 | 99.46 | 0.00E+00 |
| DFOLPJBN_00139 | 456811-457135 | 0.99 | 99.07 | 6.48E-78 |  | 465157-465481 | 0.99 | 99.07 | 6.26E-78 |  | 465240-465564 | 0.99 | 99.07 | 6.35E-78 |
| DFOLPJBN_00140 | 455712-456819 | 1.00 | 100.00 | 0.00E+00 |  | 465473-466685 | 1.00 | 99.46 | 0.00E+00 |  | 465556-466663 | 1.00 | 100.00 | 0.00E+00 |
| DFOLPJBN_00141 | 454282-454789 | 0.99 | 100.00 | 2.04E-128 |  | 467503-468010 | 0.99 | 100.00 | 1.98E-128 |  | 467586-468093 | 0.99 | 100.00 | 2.00E-128 |
| DFOLPJBN_00142 | 453626-454064 | 0.99 | 99.31 | 6.50E-107 |  | 468228-468714 | 1.00 | 90.06 | 1.28E-103 |  | 468311-468797 | 1.00 | 90.06 | 1.29E-103 |
| **DFOLPJBN_00143** | 452073-452316 | 0.99 | 100.00 | 8.53E-56 |  | **481141-482740** | **0.12** | **60.00** | **7.90E+00** |  | 470105-470348 | 0.99 | 100.00 | 8.36E-56 |
| DFOLPJBN_00144 | 451320-451833 | 0.99 | 100.00 | 2.39E-126 |  | 470516-471029 | 0.99 | 100.00 | 2.31E-126 |  | 470588-471101 | 0.99 | 100.00 | 2.34E-126 |
| DFOLPJBN_00145 | 450851-451328 | 0.99 | 100.00 | 7.67E-119 |  | 471021-471498 | 0.99 | 100.00 | 7.42E-119 |  | 471093-471570 | 0.99 | 100.00 | 7.52E-119 |
| DFOLPJBN_00146 | 450058-450700 | 1.00 | 99.06 | 1.53E-159 |  | 471649-472291 | 1.00 | 98.59 | 1.88E-159 |  | 471721-472363 | 1.00 | 97.65 | 4.27E-157 |
| **DFOLPJBN_00147** | **449245-449983** | **1.00** | **48.37** | **7.38E-72** |  | **465157-465481** | **0.30** | **30.00** | **2.00E+00** |  | **472438-473059** | **1.00** | **64.08** | **5.18E-93** |
| **DFOLPJBN_00148** | **448358-448988** | **1.00** | **81.99** | **1.18E-132** |  | **449997-452433** | **0.44** | **25.53** | **3.50E+00** |  | **450079-452515** | **0.44** | **25.53** | **3.80E+00** |
| **DFOLPJBN_00149** | 447950-448304 | 0.99 | 95.73 | 2.62E-81 |  | **473059-473656** | **0.99** | **82.91** | **2.43E-60** |  | **473364-473730** | **0.99** | **79.49** | **7.19E-69** |
| DFOLPJBN_00150 | 447589-447802 | 0.91 | 100.00 | 1.35E-49 |  | 473774-474005 | 0.97 | 100.00 | 4.98E-53 |  | 473850-474081 | 0.97 | 96.00 | 1.55E-51 |
| **DFOLPJBN_00151** | 446707-447514 | 1.00 | 97.76 | 0.00E+00 |  | **481141-482740** | **0.65** | **25.00** | **4.40E-01** |  | **474205-474961** | **0.93** | **71.49** | **3.39E-131** |
| **DFOLPJBN_00152** | 446458-446623 | 0.98 | 100.00 | 2.90E-38 |  | **483750-485538** | **0.62** | **29.41** | **3.20E+00** |  | **483520-485617** | **0.62** | **29.41** | **3.00E+00** |
| **DFOLPJBN_00153** | **444784-446455** | **0.82** | **74.63** | **0.00E+00** |  | **475374-476361** | **0.69** | **70.22** | **0.00E+00** |  | **475452-476934** | **1.00** | **77.89** | **0.00E+00** |
| DFOLPJBN_00154 | 444464-444788 | 0.99 | 100.00 | 2.61E-77 |  | 476854-477178 | 0.99 | 100.00 | 2.53E-77 |  | 476930-477254 | 0.99 | 100.00 | 2.56E-77 |
| **DFOLPJBN_00154b** | **466564-468697** | **0.70** | **28.13** | **5.80E-01** |  | **453596-455729** | **0.70** | **28.13** | **5.60E-01** |  | **453678-455811** | **0.70** | **28.13** | **5.60E-01** |
| **DFOLPJBN_00155** | **443409-444204** | **1.00** | **74.24** | **3.28E-117** |  | **481141-482740** | **0.36** | **19.72** | **2.20E-01** |  | **477514-478309** | **1.00** | **73.86** | **2.90E-116** |
| DFOLPJBN_00156 | 443198-443417 | 0.99 | 97.22 | 1.25E-47 |  | 478224-478443 | 0.99 | 98.61 | 4.54E-49 |  | 478301-478520 | 0.99 | 100.00 | 7.60E-50 |
| DFOLPJBN_00157 | 442935-443202 | 0.99 | 96.59 | 3.84E-62 |  | 478439-478706 | 0.99 | 95.46 | 1.97E-61 |  | 478516-478891 | 0.99 | 96.59 | 6.86E-62 |
| DFOLPJBN_00159 | 441971-442433 | 0.99 | 100.00 | 5.03E-114 |  | 479209-479671 | 0.99 | 98.69 | 3.92E-112 |  | 479286-479748 | 0.99 | 100.00 | 4.93E-114 |
| **DFOLPJBN_00160** | 441130-441961 | 1.00 | 100.00 | 0.00E+00 |  | **479681-480536** | **0.72** | **99.00** | **5.05E-152** |  | 479758-480589 | 1.00 | 100.00 | 0.00E+00 |
| DFOLPJBN_00161 | 440503-441115 | 1.00 | 99.51 | 1.37E-155 |  | 480526-481138 | 1.00 | 99.51 | 1.32E-155 |  | 480604-481216 | 1.00 | 99.51 | 1.34E-155 |
| DFOLPJBN_00162 | 438901-440500 | 1.00 | 100.00 | 0.00E+00 |  | 481141-482740 | 1.00 | 99.62 | 0.00E+00 |  | 481219-482818 | 1.00 | 100.00 | 0.00E+00 |
| DFOLPJBN_00163 | 438185-438905 | 1.00 | 100.00 | 0.00E+00 |  | 482736-483456 | 1.00 | 99.58 | 0.00E+00 |  | 482817-483534 | 0.99 | 100.00 | 0.00E+00 |
| DFOLPJBN_00164 | 436102-438199 | 1.00 | 100.00 | 0.00E+00 |  | 483750-485538 | 0.85 | 100.00 | 0.00E+00 |  | 483520-485617 | 1.00 | 100.00 | 0.00E+00 |
| DFOLPJBN_00165 | 435855-436086 | 0.99 | 100.00 | 6.90E-54 |  | 485554-485785 | 0.99 | 100.00 | 6.67E-54 |  | 485633-485864 | 0.99 | 100.00 | 6.76E-54 |
| DFOLPJBN_00166 | 435401-435770 | 0.99 | 99.18 | 1.19E-87 |  | 485870-486239 | 0.99 | 98.36 | 1.87E-86 |  | 485949-486318 | 0.99 | 99.18 | 1.17E-87 |
| **DFOLPJBN_00167** | 435040-435361 | 0.99 | 100.00 | 4.24E-78 |  | **485870-486239** | **0.88** | **27.66** | **5.59E-07** |  | 486358-486679 | 0.99 | 100.00 | 4.15E-78 |
| DFOLPJBN_00168 | 434486-435038 | 0.99 | 100.00 | 3.08E-139 |  | 486692-487154 | 0.83 | 99.35 | 4.91E-116 |  | 486681-487233 | 0.99 | 100.00 | 3.01E-139 |
| DFOLPJBN_00169 | 434316-434490 | 0.98 | 100.00 | 5.94E-39 |  | 487150-487324 | 0.98 | 100.00 | 5.74E-39 |  | 487229-487403 | 0.98 | 100.00 | 5.82E-39 |
| DFOLPJBN_00170 | 433291-434191 | 1.00 | 100.00 | 0.00E+00 |  | 487326-488349 | 1.00 | 99.33 | 0.00E+00 |  | 487528-488428 | 1.00 | 100.00 | 0.00E+00 |

**Table S7** Comparisons of the P-V1R1-5 proteins against the proteins identified in known CLas prophages.

| **P-V1R1-5 ID** | **P-YN-1 (Type 1)** | | |  |  | **P-GD-2 (Type 2)** | | | |  | **P-JXGC-3 (Type 3)** | | | |
| --- | --- | --- | --- | --- | --- | --- | --- | --- | --- | --- | --- | --- | --- | --- |
|  | **ID** | **Span** | **pident** | **evalue** |  | **ID** | **Span** | **pident** | **evalue** |  | **ID** | **Span** | **pident** | **evalue** |
| DFOLPJBN_00125 | SC1_gp175 | 0.16 | 40.63 | 2.70E+00 |  | SC2_gp080 | 0.30 | 20.34 | 7.50E+00 |  | PJXGC_gp08 | 0.18 | 34.29 | 8.20E+00 |
| DFOLPJBN_00126 | SC1_gp005 | 0.99 | 76.43 | 8.59E-91 |  | SC2_gp005 | 0.99 | 73.42 | 4.69E-87 |  | PJXGC_gp22 | 0.41 | 29.23 | 1.70E-02 |
| DFOLPJBN_00127 | SC1_gp010 | 1.00 | 43.33 | 4.76E-22 |  | SC2_gp255 | 1.00 | 44.44 | 4.00E-24 |  | PJXGC_gp07 | 0.37 | 34.38 | 5.60E+00 |
| DFOLPJBN_00128 | SC1_gp030 | 1.00 | 22.51 | 1.62E-30 |  | SC2_gp030 | 1.00 | 29.19 | 1.09E-96 |  | PJXGC_gp09 | 0.16 | 24.81 | 3.00E+00 |
| DFOLPJBN_00129 | SC1_gp035 | 0.87 | 25.00 | 1.04E-18 |  | SC2_gp040 | 1.00 | 24.87 | 8.71E-26 |  | PJXGC_gp07 | 0.08 | 29.03 | 6.50E+00 |
| DFOLPJBN_00130 | SC1_gp045 | 0.98 | 25.61 | 1.35E-57 |  | SC2_gp045 | 1.00 | 20.06 | 3.90E-20 |  | PJXGC_gp31 | 0.13 | 22.22 | 9.70E+00 |
| DFOLPJBN_00131 | SC1_gp050 | 0.78 | 37.61 | 3.61E-24 |  | SC2_gp065 | 0.61 | 27.17 | 7.37E-07 |  | PJXGC_gp07 | 0.36 | 31.48 | 2.00E-01 |
| DFOLPJBN_00132 | SC1_gp060 | 0.28 | 32.00 | 6.27E-25 |  | SC2_gp185 | 0.07 | 31.51 | 9.90E-01 |  | PJXGC_gp08 | 0.10 | 25.81 | 1.60E-01 |
| DFOLPJBN_00133 | SC1_gp080 | 1.00 | 38.03 | 1.18E-127 |  | SC2_gp080 | 1.00 | 29.76 | 4.90E-86 |  | PJXGC_gp11 | 0.11 | 20.31 | 1.00E+00 |
| DFOLPJBN_00134 | SC1_gp085 | 0.99 | 62.29 | 6.28E-79 |  | SC2_gp085 | 0.84 | 42.18 | 5.87E-30 |  | PJXGC_gp07 | 0.26 | 26.67 | 5.30E+00 |
| DFOLPJBN_00135 | SC1_gp090 | 0.98 | 32.45 | 2.12E-61 |  | SC2_gp090 | 0.84 | 33.22 | 5.55E-51 |  | PJXGC_gp05 | 0.13 | 26.67 | 1.90E+00 |
| DFOLPJBN_00136 | SC1_gp095 | 1.00 | 51.05 | 2.09E-75 |  | SC2_gp095 | 0.74 | 24.86 | 2.44E-10 |  | PJXGC_gp28 | 0.19 | 33.33 | 2.80E-02 |
| DFOLPJBN_00137 | SC1_gp100 | 1.00 | 67.27 | 3.69E-52 |  | SC2_gp100 | 0.76 | 43.37 | 1.58E-19 |  | PJXGC_gp23 | 0.25 | 37.04 | 3.30E+00 |
| DFOLPJBN_00138 | SC1_gp105 | 0.99 | 72.10 | 0.00E+00 |  | SC2_gp105 | 0.99 | 40.47 | 8.94E-135 |  | PJXGC_gp07 | 0.09 | 25.49 | 5.10E+00 |
| DFOLPJBN_00139 | SC1_gp050 | 0.75 | 25.93 | 4.10E+00 |  | SC2_gp040 | 0.44 | 31.92 | 3.50E-01 |  | PJXGC_gp06 | 0.23 | 28.00 | 7.70E-01 |
| DFOLPJBN_00140 | SC1_gp115 | 0.99 | 64.85 | 2.91E-180 |  | SC2_gp115 | 0.99 | 60.44 | 5.81E-147 |  | PJXGC_gp11 | 0.75 | 68.23 | 1.56E-141 |
| DFOLPJBN_00141 | SC1_gp120 | 0.88 | 37.84 | 2.86E-26 |  | SC2_gp120 | 0.88 | 37.84 | 2.73E-26 |  | PJXGC_gp14 | 0.88 | 37.58 | 1.03E-24 |
| DFOLPJBN_00142 | SC1_gp100 | 0.26 | 28.95 | 7.10E-01 |  | SC2_gp105 | 0.17 | 44.00 | 9.50E-01 |  | PJXGC_gp09 | 0.36 | 33.96 | 1.80E+00 |
| DFOLPJBN_00143 | SC1_gp140 | 0.19 | 46.67 | 7.50E+00 |  | SC2_gp080 | 0.56 | 28.89 | 1.50E+00 |  | PJXGC_gp18 | 0.19 | 46.67 | 5.10E+00 |
| DFOLPJBN_00144 | SC1_gp060 | 0.73 | 23.39 | 9.40E-01 |  | SC2_gp030 | 0.38 | 26.15 | 6.00E+00 |  | PJXGC_gp29 | 0.37 | 31.25 | 1.90E-01 |
| DFOLPJBN_00145 | SC1_gp045 | 0.92 | 19.86 | 2.50E+00 |  | SC2_gp265 | 0.43 | 24.64 | 1.70E-01 |  | PJXGC_gp08 | 0.19 | 29.03 | 3.60E+00 |
| DFOLPJBN_00146 | SC1_gp185 | 0.18 | 26.32 | 1.30E+00 |  | SC2_gp040 | 0.21 | 34.09 | 1.30E-01 |  | PJXGC_gp16 | 0.19 | 31.71 | 1.20E-01 |
| DFOLPJBN_00147 | SC1_gp125 | 0.80 | 52.50 | 2.61E-59 |  | SC2_gp125 | 0.81 | 51.55 | 3.11E-53 |  | PJXGC_gp15 | 0.78 | 56.13 | 1.15E-57 |
| DFOLPJBN_00148 | SC1_gp130 | 0.87 | 45.11 | 6.57E-50 |  | SC2_gp130 | 0.34 | 39.73 | 7.08E-14 |  | PJXGC_gp16 | 0.34 | 36.99 | 1.52E-13 |
| DFOLPJBN_00149 | SC1_gp135 | 1.00 | 62.10 | 1.51E-54 |  | SC2_gp135 | 1.00 | 62.10 | 1.44E-54 |  | PJXGC_gp17 | 1.00 | 62.10 | 1.03E-54 |
| DFOLPJBN_00150 | SC1_gp140 | 0.83 | 56.25 | 2.27E-24 |  | SC2_gp140 | 0.83 | 56.25 | 2.16E-24 |  | PJXGC_gp18 | 0.83 | 56.25 | 1.55E-24 |
| DFOLPJBN_00151 | SC1_gp200 | 0.65 | 52.30 | 3.57E-55 |  | SC2_gp200 | 0.65 | 52.30 | 3.41E-55 |  | PJXGC_gp29 | 0.65 | 50.00 | 2.94E-53 |
| DFOLPJBN_00152 | SC1_gp155 | 0.40 | 36.36 | 2.30E-01 |  | SC2_gp080 | 0.49 | 22.22 | 2.90E-01 |  | PJXGC_gp09 | 0.47 | 30.77 | 1.50E-02 |
| DFOLPJBN_00153 | SC1_gp025 | 0.87 | 25.84 | 4.67E-17 |  | SC2_gp020 | 0.36 | 24.17 | 6.70E-01 |  | PJXGC_gp01 | 0.11 | 36.07 | 7.90E-01 |
| DFOLPJBN_00154 | SC1_gp160 | 1.00 | 72.80 | 2.15E-57 |  | SC2_gp160 | 1.00 | 72.80 | 2.05E-57 |  | PJXGC_gp22 | 0.99 | 71.96 | 5.46E-49 |
| DFOLPJBN_00154b | SC1_gp220 | 0.41 | 36.84 | 5.50E+00 |  | SC2_gp220 | 0.41 | 36.84 | 5.20E+00 |  | PJXGC_gp33 | 0.41 | 36.84 | 3.50E+00 |
| DFOLPJBN_00155 | SC1_gp170 | 0.55 | 42.20 | 1.01E-17 |  | SC2_gp170 | 0.55 | 42.20 | 1.58E-17 |  | PJXGC_gp24 | 0.56 | 43.24 | 9.81E-19 |
| DFOLPJBN_00156 | SC1_gp235 | 0.48 | 34.29 | 2.60E+00 |  | SC2_gp195 | 0.40 | 37.93 | 8.50E+00 |  | PJXGC_gp14 | 0.34 | 32.00 | 4.20E+00 |
| DFOLPJBN_00157 | SC1_gp025 | 0.37 | 33.33 | 7.70E+00 |  | SC2_gp180 | 0.89 | 70.89 | 6.64E-36 |  | PJXGC_gp29 | 0.76 | 23.53 | 2.30E+00 |
| DFOLPJBN_00159 | SC1_gp195 | 0.69 | 23.59 | 5.48E-05 |  | SC2_gp195 | 0.69 | 23.59 | 4.89E-05 |  | PJXGC_gp28 | 0.69 | 23.59 | 3.61E-05 |
| DFOLPJBN_00160 | SC1_gp115 | 0.37 | 23.30 | 6.80E-01 |  | SC2_gp115 | 0.37 | 23.30 | 8.00E-01 |  | PJXGC_gp11 | 0.37 | 22.33 | 4.70E-01 |
| DFOLPJBN_00161 | SC1_gp215 | 0.41 | 22.62 | 2.40E-01 |  | SC2_gp215 | 0.41 | 22.62 | 2.70E-01 |  | PJXGC_gp32 | 0.41 | 22.62 | 2.90E-01 |
| DFOLPJBN_00162 | SC1_gp220 | 0.10 | 27.45 | 7.90E+00 |  | SC2_gp165 | 0.17 | 27.17 | 1.50E+00 |  | PJXGC_gp07 | 0.25 | 23.66 | 1.00E-02 |
| DFOLPJBN_00163 | SC1_gp165 | 0.43 | 24.27 | 4.10E+00 |  | SC2_gp165 | 0.43 | 25.24 | 1.80E+00 |  | PJXGC_gp23 | 0.43 | 24.27 | 4.30E+00 |
| DFOLPJBN_00164 | SC1_gp045 | 0.03 | 36.36 | 1.70E+00 |  | SC2_gp230 | 0.04 | 36.00 | 1.90E+00 |  | PJXGC_gp03 | 0.07 | 27.45 | 9.10E+00 |
| DFOLPJBN_00165 | SC1_gp165 | 0.48 | 27.03 | 2.80E+00 |  | SC2_gp165 | 0.39 | 36.67 | 7.80E+00 |  | PJXGC_gp10 | 0.56 | 37.21 | 5.40E-02 |
| DFOLPJBN_00166 | SC1_gp225 | 0.92 | 24.78 | 5.57E-05 |  | SC2_gp225 | 0.92 | 24.78 | 5.31E-05 |  | PJXGC_gp34 | 0.92 | 24.78 | 3.81E-05 |
| DFOLPJBN_00167 | SC1_gp225 | 0.91 | 46.39 | 2.57E-28 |  | SC2_gp225 | 0.91 | 46.39 | 2.45E-28 |  | PJXGC_gp34 | 0.91 | 46.39 | 1.76E-28 |
| DFOLPJBN_00168 | SC1_gp230 | 0.97 | 54.19 | 4.25E-67 |  | SC2_gp230 | 0.95 | 71.26 | 4.28E-93 |  | PJXGC_gp35 | 0.95 | 71.26 | 1.24E-92 |
| DFOLPJBN_00169 | SC1_gp015 | 0.84 | 20.41 | 9.70E+00 |  | SC2_gp125 | 0.47 | 37.04 | 1.10E+00 |  | PJXGC_gp30 | 0.31 | 27.78 | 4.80E+00 |
| DFOLPJBN_00170 | SC1_gp045 | 0.42 | 24.60 | 7.20E+00 |  | SC2_gp120 | 0.17 | 25.00 | 2.80E-01 |  | PJXGC_gp07 | 0.22 | 28.36 | 2.30E-01 |

**Table S8** Information on the *Ca*. Liberibacter samples used for the P-V1R1-5 prophage variability and distribution analysis (Table 1).

| **Sample ID** | **Origin** | **Species** | **Reference** | **P-V1R1-5 region amplifications with indicated primers** | | | | | | | | | | **PCR profile** |
| --- | --- | --- | --- | --- | --- | --- | --- | --- | --- | --- | --- | --- | --- | --- |
|  |  |  |  | **VA** | **VB** | **VC** | **VD** | **VF** | **VG** | **VH** | **VJ** | **VO** | **VQ** |  |
| HLB19-471 | Madagascar | CLaf | This study | 1 | 1 | 1 | 1 | 0 | 0 | 1 | 1 | 1 | 1 | 4 |
| HLB19-481 | Madagascar | CLaf | This study | 1 | 1 | 1 | 1 | 1 | 0 | 1 | 1 | 1 | 1 | 2 |
| HLB19-500 | Madagascar | CLaf | This study | 1 | 1 | 1 | 1 | 1 | 0 | 1 | 1 | 1 | 1 | 2 |
| HLB19-503 | Madagascar | CLaf | This study | 1 | 1 | 1 | 1 | 1 | 0 | 1 | 1 | 1 | 1 | 2 |
| HLB19-529 | Madagascar | CLaf | This study | 1 | 1 | 1 | 1 | 1 | 0 | 1 | 1 | 1 | 1 | 2 |
| HLB20-174 | Madagascar | CLaf | This study | 1 | 1 | 1 | 1 | 1 | 0 | 1 | 1 | 1 | 1 | 2 |
| HLB20-180 | Madagascar | CLaf | This study | 1 | 1 | 1 | 1 | 1 | 0 | 1 | 1 | 1 | 1 | 2 |
| HLB18-149 | Réunion | CLas | https://doi.org/10.1111/eva.70053 | 1 | 1 | 1 | 1 | 1 | 0 | 1 | 1 | 1 | 1 | 2 |
| HLB18-183 | Réunion | CLas | https://doi.org/10.1111/eva.70053 | 1 | 1 | 1 | 1 | 1 | 0 | 1 | 1 | 1 | 1 | 2 |
| HLB18-233 | Réunion | CLas | https://doi.org/10.1111/eva.70053 | 1 | 1 | 1 | 1 | 1 | 0 | 1 | 1 | 1 | 1 | 2 |
| HLB18-243 | Réunion | CLas | https://doi.org/10.1111/eva.70053 | 1 | 1 | 1 | 1 | 1 | 0 | 1 | 1 | 1 | 1 | 2 |
| HLB19-011 | Réunion | CLas | https://doi.org/10.1111/eva.70053 | 1 | 1 | 1 | 1 | 1 | 0 | 1 | 1 | 1 | 1 | 2 |
| HLB19-050 | Réunion | CLas | https://doi.org/10.1111/eva.70053 | 1 | 1 | 1 | 1 | 1 | 0 | 1 | 1 | 1 | 1 | 2 |
| HLB19-410 | Réunion | CLas | https://doi.org/10.1111/eva.70053 | 1 | 1 | 1 | 1 | 1 | 0 | 1 | 1 | 1 | 1 | 2 |
| HLB19-419 | Réunion | CLas | https://doi.org/10.1111/eva.70053 | 1 | 1 | 1 | 1 | 1 | 0 | 1 | 1 | 1 | 1 | 2 |
| HLB19-573 | Réunion | CLas | https://doi.org/10.1111/eva.70053 | 1 | 1 | 1 | 1 | 1 | 0 | 1 | 1 | 1 | 1 | 2 |
| HLB19-583 | Réunion | CLas | https://doi.org/10.1111/eva.70053 | 0 | 0 | 0 | 0 | 0 | 0 | 0 | 0 | 0 | 0 | - |
| HLB19-624 | Réunion | CLas | https://doi.org/10.1111/eva.70053 | 1 | 1 | 1 | 1 | 1 | 1 | 1 | 1 | 1 | 1 | 1 |
| HLB20-071 | Réunion | CLas | https://doi.org/10.1111/eva.70053 | 1 | 1 | 1 | 1 | 1 | 0 | 1 | 1 | 1 | 1 | 2 |
| HLB20-367 | Réunion | CLas | https://doi.org/10.1111/eva.70053 | 0 | 1 | 1 | 1 | 1 | 0 | 1 | 1 | 1 | 1 | 5 |
| HLB20-405 | Réunion | CLas | https://doi.org/10.1111/eva.70053 | 1 | 1 | 1 | 1 | 1 | 1 | 1 | 1 | 1 | 1 | 1 |
| HLB21-037 | Réunion | CLas | https://doi.org/10.1111/eva.70053 | 1 | 1 | 1 | 1 | 1 | 1 | 1 | 1 | 1 | 1 | 1 |
| HLB21-040 | Réunion | CLas | https://doi.org/10.1111/eva.70053 | 1 | 1 | 1 | 1 | 1 | 1 | 1 | 1 | 1 | 1 | 1 |
| HLB21-050 | Réunion | CLas | https://doi.org/10.1111/eva.70053 | 0 | 0 | 0 | 0 | 0 | 0 | 0 | 0 | 0 | 0 | - |
| HLB21-080 | Réunion | CLas | https://doi.org/10.1111/eva.70053 | 1 | 1 | 1 | 1 | 1 | 0 | 1 | 1 | 1 | 1 | 2 |
| HLB21-086 | Réunion | CLas | https://doi.org/10.1111/eva.70053 | 0 | 0 | 0 | 0 | 0 | 0 | 0 | 0 | 0 | 0 | - |
| HLB21-099 | Réunion | CLas | https://doi.org/10.1111/eva.70053 | 1 | 1 | 1 | 1 | 1 | 1 | 1 | 1 | 1 | 1 | 1 |
| HLB21-102 | Réunion | CLas | https://doi.org/10.1111/eva.70053 | 1 | 1 | 1 | 1 | 1 | 1 | 1 | 1 | 1 | 1 | 1 |
| HLB21-138 | Réunion | CLas | https://doi.org/10.1111/eva.70053 | 1 | 1 | 1 | 1 | 1 | 1 | 1 | 1 | 1 | 1 | 1 |
| HLB21-157 | Réunion | CLas | https://doi.org/10.1111/eva.70053 | 1 | 1 | 1 | 1 | 0 | 0 | 1 | 1 | 1 | 1 | 4 |
| HLB21-328 | Réunion | CLas | https://doi.org/10.1111/eva.70053 | 1 | 1 | 1 | 1 | 1 | 1 | 1 | 1 | 1 | 1 | 1 |
| HLB21-332 | Réunion | CLas | https://doi.org/10.1111/eva.70053 | 1 | 1 | 1 | 1 | 1 | 0 | 1 | 1 | 1 | 1 | 2 |
| HLB21-340 | Réunion | CLas | https://doi.org/10.1111/eva.70053 | 1 | 0 | 1 | 1 | 1 | 1 | 1 | 1 | 1 | 1 | 3 |
| HLB22-009 | Réunion | CLas | https://doi.org/10.1111/eva.70053 | 0 | 0 | 0 | 0 | 0 | 0 | 0 | 0 | 0 | 0 | - |
| HLB22-027 | Réunion | CLas | https://doi.org/10.1111/eva.70053 | 1 | 1 | 1 | 1 | 1 | 0 | 1 | 1 | 1 | 1 | 2 |
| HLB23-032 | Réunion | CLaf | https://doi.org/10.1111/eva.70053 | 1 | 1 | 1 | 1 | 1 | 0 | 1 | 1 | 1 | 1 | 2 |
| HLB23-033 | Réunion | CLaf | https://doi.org/10.1111/eva.70053 | 1 | 1 | 1 | 1 | 1 | 0 | 1 | 1 | 1 | 1 | 2 |
| HLB23-035 | Réunion | CLaf | https://doi.org/10.1111/eva.70053 | 1 | 1 | 1 | 1 | 1 | 0 | 1 | 1 | 1 | 1 | 2 |
| HLB23-036 | Réunion | CLaf | https://doi.org/10.1111/eva.70053 | 1 | 1 | 1 | 1 | 1 | 0 | 1 | 1 | 1 | 1 | 2 |

**Table S9** CLas titer in the 29 psyllid samples used for sequencing.

| **Sample ID** | **Mean Ct** | **DNA concentration (ng/µl)** | **Total DNA (ng)** |
| --- | --- | --- | --- |
| 2 | 18.23 | 2.76 | 165.60 |
| 3 | 20.76 | 0.90 | 54.00 |
| 4 | 17.32 | 0.98 | 62.72 |
| 5 | 18.31 | 0.60 | 36.00 |
| 10 | 18.60 | 1.86 | 111.60 |
| 11 | 15.94 | 2.50 | 150.00 |
| 12a | 16.52 | 2.06 | 123.60 |
| 12b | 16.25 | 1.47 | 88.20 |
| 13a | 19.02 | 0.48 | 28.80 |
| 13b | 17.08 | 0.61 | 36.60 |
| 14 | 17.41 | 0.81 | 51.84 |
| 16 | 17.63 | 1.04 | 66.56 |
| 17 | 18.74 | 5.36 | 343.04 |
| 18 | 19.29 | 3.06 | 183.60 |
| 22 | 16.64 | 0.88 | 52.80 |
| 23 | 16.90 | 1.36 | 81.60 |
| 26 | 20.26 | 3.78 | 226.80 |
| 31 | 18.26 | 0.97 | 58.20 |
| 32 | 18.88 | 1.16 | 69.60 |
| 33 | 18.19 | 1.25 | 75.00 |
| 35 | 20.40 | 0.80 | 48.00 |
| 36 | 18.30 | 1.68 | 100.80 |
| 37 | 18.74 | 1.43 | 85.80 |
| 38 | 19.31 | 0.67 | 40.20 |
| 39 | 18.53 | 2.14 | 128.40 |
| 40 | 20.32 | 0.58 | 34.80 |
| 45 | 18.00 | 1.81 | 108.60 |
| 47 | 19.53 | 1.06 | 63.60 |
| 48 | 19.00 | 0.40 | 24.00 |
